# Spontaneous Salivation

**Published:** 1863-11

**Authors:** 


### CORRESPONDENCE.

### SPONTANEOUS SALIVATION.

Editor Medical and Surgical Journal:

In the last number of the *Journal* I notice an article on "Spontaneou Salivation; a Suggestion," which to me appears so wonderfully suggestive, that when I read it I felt induced to lay down my meerschaum and write a short discourse, taking said article for my text. I will commence by asserting my belief that the Surgeon-General may be more than half right in withholding such dangerous medicines as calomel and tartar emetic, from the supply table, for the reason that many who were fortunate enough through political or other powerful influences to obtain medical appointments in the volunteer service, knew little or nothing, practically, of the effects of those powerful drugs upon the human system, and hence the reason that a routine practice of making prescriptions, into which Hydrargyrum in some form entered largely, prevailed; and it is not strange that with the use of vinegar and other acids with which it would come in contact in the stomach, changing the mild chloride to the bi-chloride, that salivation would follow and that frequently.

It cannot be denied that there is a recklessness in the army, not only in prescriptions but of human life generally. Who would like to trust the physician with the care of his loved ones at home, who would make a prescription founded upon the following short scientific examination:

What is the matter with you? How are your bowels? Let me see your tongue.

Pilula hydrargyri et morphia. No matter what the complaint, the same prescription for all.

Or would one who would himself take seventy grains of sulph. quinine at a single dose, be any more safe. This I have seen and was not surprised to see the same surgeon, a few days after on his way to Washington, sick and his regiment deprived of his (valuable?) services on the eve of, and during a series of battles. But I am wandering from my text. The article says "While serving with the 21st Regiment in August, 1862, I met several cases of spontaneous salivation, and as they served to prove to me, at least, that the disease was not a myth, and that it might exist in the army, I am inclined to the belief that it may have often been mistaken for the mercurial affection." Who ever supposed that the disease described by authors as Spontaneous Salivation was a myth? If pregnant women and teething children composed the army, and particularly the 21st Regiment, it would not surprise me that the doctor should meet with "*several cases in that Regiment in August*, 1862." But as that Regiment was composed of full-grown men (in whom the disease is certainly rare), I cannot but distrust the doctor's diagnosis. It is unfortunate, *perhaps*, for the cause of science and the unfortunate surgeons of the army, that the Doctor kept no records and has to rely upon memory for such an important discovery; one that unfolds all the mystery of the Surgeon-General's order in regard to Calomel and Tartar Emetic. It is all explained now in the wonderful discovery of "Spontaneous Salivation." Let us take a look at the "cases:"

1st, "In August, 1862 . . . . I discovered symptoms of severe salivation in a patient in the regimental hospital. As I had been giving him some calomel, I of course attributed it to that, and such was the view of Dr. Wilcox. My only difficulty was to account for so severe symptoms from such a small quantity of the drug as he had taken." Now here is a case of severe salivation following the administration of calomel, believed to be such at the time by the surgeon giving it, and that opinion confirmed by Dr. Wilcox, and now cited by our learned friend, as an example of "spontaneous salivation." The profession ought to be forever grateful for such an irrefragible argument.

Case 2.—"A few days later I found the same affection in a man who had taken no mercury at all," (queer). This is all said of case 2. Now, I am disposed to take this statement with many grains of allowance. Many of the officers and men carried medicines of their own, and may have taken it unknown to the surgeon. And where there is so much shirking duty as there is in the army, they would not be likely to tell if they had obtained the medicine and taken it. It is a pity that the Doctor's memory does not extend to the other four cases, as he says there were "about half-a-dozen cases in the 21st Regiment, in the month of August." That regiment, on the first of August, 1862, may have numbered six hundred men. That would be one per cent affected with "spontaneous salivation," some, at least, of whom had taken calomel. This would give us, if we have 500,000 men in the field, 5,000 salivated men with or without calomel in a single month. There is no good reason for supposing that the disease was confined to the 21st regiment. This statement being true, is it any wonder the Surgeon-General opened his eyes to the matter, and cause of all this salivation?

To detect the difference between mercurial and spontaneous salivation would not, I fancy, be very difficult for an experienced physician. Hence in my mind arise serious doubts as to the correctness of the Doctor's memory or conclusions, for this reason. On the 9th of August, 1862, every man in the 21st regiment, sick and unfit for active duty, was sent to the hospital, some 40 or more. At daylight the regiment marched and participated in the Pope campaign, during which time, while marching and fighting, there must have been little time for observation, for the man that could not march and fight would of necessity receive very slight attention from the surgeons, for the race was swift and dangerous. To establish his "theory" the Doctor very conveniently for him, but equally unfortunately for his case, "puts mercury out of the question, even in the cases of those who had taken it." and innocently thinks there was no mistake in his diagnosis. It is from no "pique" that I have noticed in this frank manner the Doctor's paper. I have ever looked upon him as one destined to make his mark in the profession; and this notice will, I trust, be met by him in the same spirit, and if it should unfortunately cause irritation and the "other plans of treatment" should fail to soothe it, that the remedy found "most effectual" will cure the wound as readily as it did "spontaneous salivation."

By what process of reasoning a surgeon can "put mercury out of the question even in the case of those who had taken it," is certainly too much for my poor comprehension. Nor does it depend so much upon idiosyncracy of constitution, as upon the presence of an excess of acids. "Two grains of calomel may, and has been known to produce severe salivation when followed by dilute sulph. acid. The acids naturally contained in the stomach are the muriatic and acetic, and a grain or two of calomel or a few grains of blue mass would be sufficient to cause serious salivation, if thus changed into the bi-chloride."*

In conclusion permit me to say, that the "obloquy heaped upon the heads of some army surgeons cannot be so easily averted, even were it generally known that salivation is not always a sign of over-dosing by mercury."

MEDICUS.

* Braithwaite, Part I, page 45.

STURGIS, MICH., October 21, 1863.

Editor of Med. and Surg. Journal, Buffalo, N. Y.:

Dear Doctor:—In my communication in October number of the *Journal*, upon "Spotted Fever," I find two errors. On page 83, 3d line, for "characteristic symptoms," read "characteristic *eruption*." On page 84, 13th and 15th lines, for "1853" read "1863."

I will endeavor to send you another communication for the December number.

Yours, truly, E. W. JENKS.

